# Prevalence of and Factors Associated with Human Cysticercosis in 60 Villages in Three Provinces of Burkina Faso

**DOI:** 10.1371/journal.pntd.0004248

**Published:** 2015-11-20

**Authors:** Hélène Carabin, Athanase Millogo, Assana Cissé, Sarah Gabriël, Ida Sahlu, Pierre Dorny, Cici Bauer, Zekiba Tarnagda, Linda D Cowan, Rasmané Ganaba

**Affiliations:** 1 Department of Biostatistics and Epidemiology, College of Public Health, Oklahoma University Health Sciences Center, Oklahoma City, Oklahoma, United States of America; 2 Centre Hospitalier Universitaire Souro Sanou, Bobo-Dioulasso, Burkina Faso; 3 Institut de Recherche et des Sciences de la Santé, Bobo Dioulasso, Burkina Faso; 4 Department of Biomedical Sciences, Institute of Tropical Medicine, Antwerp, Belgium; 5 Department of Epidemiology, Brown School of Public Health, Providence, Rhode Island, United States of America; 6 Population Studies and Training Center, Brown University, Providence, Rhode Island, United States of America; 7 Department of Biostatistics, Brown School of Public Health, Providence, Rhode Island, United States of America; 8 Institut de Recherche en Sciences de la Santé, Bobo-Dioulasso, Burkina Faso; 9 Agence de Formation de Recherche et d'Expertise en Santé pour l’Afrique (AFRICSanté), Bobo-Dioulasso, Burkina Faso; The First Affiliated Hospital of Xinjiang Medical University, CHINA

## Abstract

**Background:**

*Taenia solium*, a zoonotic infection transmitted between humans and pigs, is considered an emerging infection in Sub-Saharan Africa, yet individual and community-level factors associated with the human infection with the larval stages (cysticercosis) are not well understood. This study aims to estimate the magnitude of association of individual-level and village-level factors with current human cysticercosis in 60 villages located in three Provinces of Burkina Faso.

**Methodology/Principal Findings:**

Baseline cross-sectional data collected between February 2011 and January 2012 from a large community randomized-control trial were used. A total of 3609 individuals provided serum samples to assess current infection with cysticercosis. The association between individual and village-level factors and the prevalence of current infection with cysticercosis was estimated using Bayesian hierarchical logistic models. Diffuse priors were used for all regression coefficients. The prevalence of current cysticercosis varied across provinces and villages ranging from 0% to 11.5%. The results obtained suggest that increased age, being male and consuming pork as well as a larger proportion of roaming pigs and percentage of sand in the soil measured at the village level were associated with higher prevalences of infection. Furthermore, consuming pork at another village market had the highest increased prevalence odds of current infection. Having access to a latrine, living in a household with higher wealth quintiles and a higher soil pH measured at the village level decreased the prevalence odds of cysticercosis.

**Conclusions/Significance:**

This is the first large-scale study to examine the association between variables measured at the individual-, household-, and village-level and the prevalence odds of cysticercosis in humans. Factors linked to people, pigs, and the environment were of importance, which further supports the need for a One Health approach to control cysticercosis infection.

## Author Summary

*Taenia solium* is an infection that is transmitted between pigs and humans. Humans may get infected with the larvae of *Taenia solium*, which results in cysticercosis, an infection common in pig farming communities where there is poor sanitation and free roaming pigs. Most published studies on this infection have included less participants covering a restricted geographic area, thereby resulting in a limited understanding of the important risk factors for infection. Our study aimed to examine important individual-, household- and village-level characteristics associated with current infection using baseline data from 3609 participants living in 60 villages across three provinces in Burkina Faso. Blood samples from village participants were taken to assess whether they were infected with cysticercosis. We found that eating pork, especially in other village’s markets, being older and male, living in a poorer household, not having access to a latrine, and living in a village where a larger percentage of pigs are left roaming were associated with infection. Soil pH and composition may also play a role in infection. Our results suggest that interventions that include human and veterinary health as well as environmental components should be considered to effectively control cysticercosis in such settings.

## Introduction

*Taenia solium* cysticercosis, a zoonotic infection transmitted between humans and pigs, is considered as an emerging infection in Sub-Saharan Africa. In the Sahel and West Africa region, the pig population has increased by 23% between 1985 and 2005, the largest increase in all animal populations during that period [1]. The pig population more than doubled in Burkina Faso between 2000 and 2008 [2]. Nearly 98% of pigs in the country are raised in a traditional manner in small holder farming communities, and left free roaming to fetch their own food [2]. The Joint Monitoring Programme (UNICEF/WHO) estimated the percentage of improved sanitation in rural Burkina Faso to be 8% at the end of 2008, far below the target of 54% set for 2015 by the Burkina Faso National Program for Drinking Water Supply and Sanitation [3]. The increase in primarily traditionally raised pig population and the lack of improvement in sanitation in rural areas are ideal conditions for the spread and maintenance of *T*. *solium* infection in humans and pigs. This is likely to have great consequences on public health since humans, when infected with the larval stages of the infection, may develop neurocysticercosis (NCC). NCC is a preventable cause of multiple neurological manifestations including epilepsy, severe chronic headaches, and focal deficits, to name but a few [4–6].

Serological studies using tests to detect antigens or antibodies have demonstrated the presence of the infection in humans [7–9, reviewed by 10] and pigs (reviewed by [11]) in several countries of West Africa, but with some large variation in estimates from country to country and from village to village within countries. Part of the variation could be explained by the use of antibody-detecting tests such as the EITB [12], which measure past exposure to and current infection with living metacestodes, and antigen-detecting tests [13,14], which measure current infection with living metacestodes. For example, in Burkina Faso, no human was found to have a strong positive AgELISA result in a village where very few pigs were present, while the prevalence of strong positives in humans was between 1.4% and 10.3% and in pigs between 32.5% and 39.6% in two villages where pigs were raised [15,16].

Previous studies conducted in West Africa have included a small number of participants or of villages, limiting the ability to detect associations, and especially weaker ones, between village-level as well as individual-level variables and infection. A better understanding of individual and community-level factors associated with infection is essential to developing effective control strategies to reduce the burden of this devastating disease.

The aim of this study is to estimate the magnitude of association of individual-, household- and village-level factors with current human cysticercosis infection in 60 villages located in three Provinces of Burkina Faso.

## Methods

### Study design

This study reports on the baseline cross-sectional component of a large community randomized-control trial aimed at estimating the effectiveness of an educational package on reducing the cumulative incidence of current infection of human and porcine cysticercosis. The baseline cross-sectional study took place between February 2011 and January 2012.

### Study area and selection of study villages

**Fig 1:**
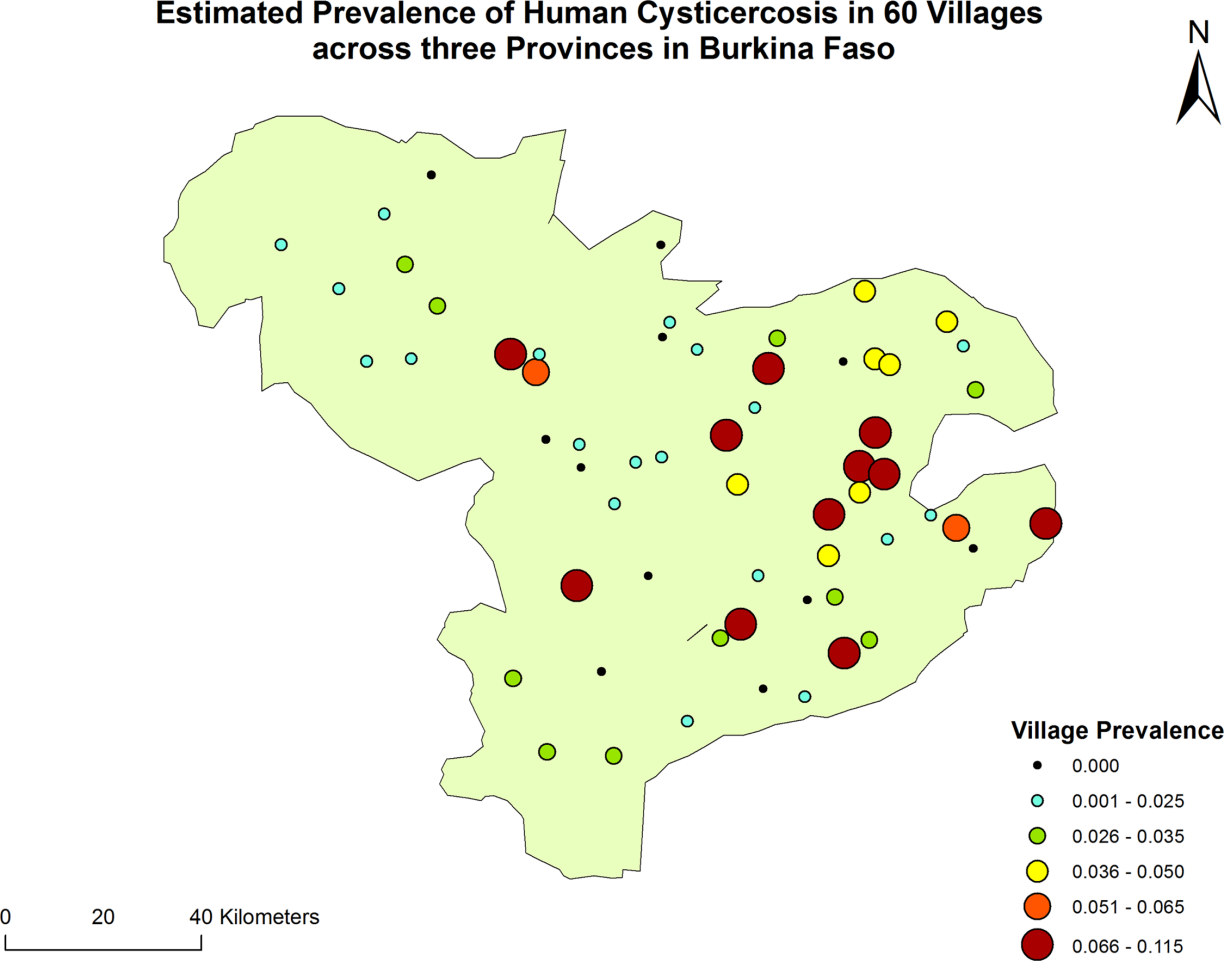
Location of the 60 participating villages in three Provinces of Burkina Faso. The estimated village prevalence of human cysticercosis for the 60 villages is presented in color-coded circles.

The provinces of Nayala (Region of Boucle du Mouhon), Boulkiemdé and Sanguié (Region of Centre-Ouest) were selected for inclusion in the study. Boulkiemdé and Sanguié are among the provinces with the largest number of pigs in the country (191,438 and 145,923 heads respectively in 2010 [17]. Nayala has an estimated pig population of 41,521 and was selected because of local reports that humans tend to defecate in pigsties where most pigs are kept during the rainy season and because it was neighbouring the other two provinces. In each province, all departments where pigs were raised (30 of 31 departments) were selected. Within each department, two villages meeting the eligibility criteria were selected at random. The exclusion criteria were: being located on a National or Provincial road; being the Chef-Lieu (Capital) of the Region or of the Province; being located within 20 km of Koudougou or Ouagadougou. For the purpose of the parent community-based trial, the inclusion criteria were: having a population of at least 1000 people at the 2006 census; being present on the map from the *Institut Géographique du Burkina* (from the year 2000); being separated from another participating village by at least 5 kilometers. A third village meeting all the eligibility criteria was also selected at random in each department as a replacement if the village initially sampled were to be found not eligible during the field visit (for example, too few households raising pigs, refusal from the village leaders which happened in one instance). Only one village met the eligibility criteria in the Department of Zamo (Province of Sanguié). A village located in the Department of Pouni but right on the border with Zamo was selected as the second village for that Department. The location of all participating villages is illustrated on Fig 1.

### Census of concessions in each village

A *concession* (compound) is defined here as a group of households living in a residential development, often fenced, where the authority of a concession chief is recognized. A *household* is defined as a socio-economic unit where members live together, share resources and satisfy the food and other essential needs of all members. In general, a household includes a man, his wife or wives, their children, and any other person who shares resources with other members of the household.

In each village, the research team of five or six interviewers started by visiting the concession of the village Chief. The field team moved counter clockwise to enumerate each concession. In each concession, starting on the right from the entrance, the field team enumerated each household, as well as the number of members and if someone raised piglets less than 12 months old or reproductive sows in each household.

### Sampling of concessions

**Fig 2:**
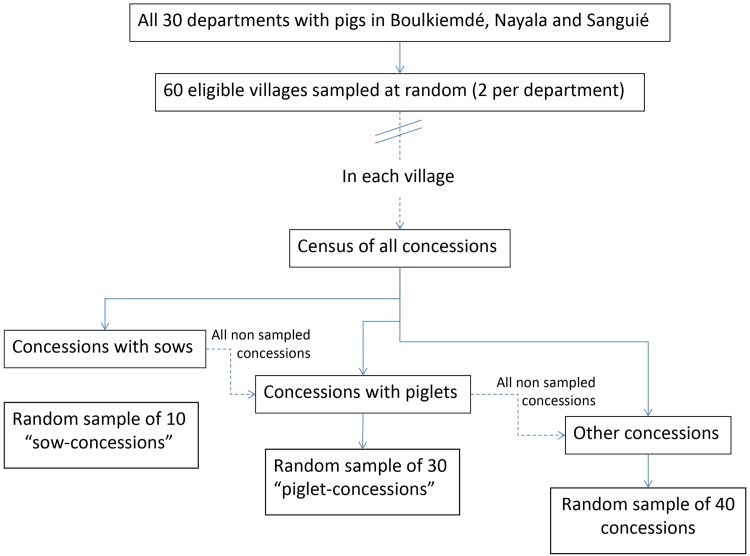
Flowchart of sampling strategy of concessions in each village.

A stratified random sampling approach was adopted to select concessions. The strata were the presence of reproductive sows, piglets, or no pigs. First, the list of all concessions where there was at least one household raising sows was made and corresponding concession numbers were placed in a bag. A village member was asked to sample 10 numbers from the bag. If 10 or less concessions were raising sows, all of those concessions were invited to participate. Next, the numbers of all concessions where at least one household raised piglets aged 12 months or younger were placed in a bag, including concessions where sows were also raised but that were not selected in the previous step. A village member was asked to sample 30 additional numbers from the bag. If 30 or less concessions raised piglets less than 12 months of age, all of those concessions were invited to participate. Lastly, the numbers of all concessions not yet sampled were placed in a bag. A village member was asked to sample an additional 40 numbers. This resulted in the sampling of a total of 80 concessions in each village, with at least 10 concessions with sows and at least 30 concessions with piglets aged less than 12 months, except in a few villages in Nayala where there were less than 40 concessions raising pigs in the whole village. Fig 2 summarizes the sampling strategy for the concessions. Only one Chief of a concession refused participation in a village of Boulkiemdé and was replaced by another concession in the village.

### Sampling of households

Once the Chief of a sampled concession gave his/her consent to participate in the study, he was asked to enumerate all households in the concession with the names of their Heads. Numbers corresponding to the households were placed in a bag and the chief of the concession was asked to sample one number from the bag, or, if there was only one household in the concession, that household was selected. The Chief of the selected household was asked for his consent to participate in the study and to enumerate all members of his household. Only one Chief of household refused participation in Boulkiemdé and asked that his father’s household be sampled instead of his, as a mark of respect for his elder. All household Chiefs consented to participate.

### Sampling of participants

Each eligible household member was allocated a number. The inclusion criteria for participants were that they were at least 5 years old, had lived in the village for at least 12 months and were not planning to move in the next three years. The head of the household was asked to pick a number from the bag. The person with the corresponding number was asked for his/her consent to participate in the study which included a screening questionnaire on epilepsy, progressively worsening severe chronic headaches as well as a blood sample to test for the presence of antigens to the larval stages of cysticercosis. Participation in the study involved answering the screening questionnaire and having blood collected three times in a three-year period for the purpose of the parent community-based randomized controlled trial. The sampling process (starting with the concession sampling) was repeated until 60 people consented to being screened and having blood collected three times over three years duration of the randomized trial. If the sampled person refused to consent to the serological component of the study, s/he was asked for his/her consent to participate in the screening-only study for a maximum of 20 screening-only participants per village. If the sampled individual did not consent to either the serological or screening study, the head of the household was asked to sample another number from the bag for that household until a consenting individual was found. There were a few refusals to the serological participation, and all cases were replaced as described above. No one refused to participate to the neurological component of the study.

### Questionnaires

Each consenting participant was asked to answer an individual questionnaire including questions about socio-demographic factors, pork consumption behavior, knowledge of *T*. *solium* infection and life cycle, and screening questions on seizures, epilepsy, and severe chronic headaches. Since the focus of the current study is to estimate the magnitude of association between potential risk factors and current cysticercus infection, results from screening for epilepsy and progressively worsening severe chronic headaches are not reported. In addition, the Chief of each participating household was asked to list assets owned by members of the household (ie bicycles, carts, livestock, etc). The mother of each household was asked questions about preparation of pork and access and use of latrines by members of the household. The building material of the house’s roof, floor and walls was also measured at that time. In each village, the owners of at most 10 sows and 30 piglets aged less than 12 months were interviewed regarding their pig management practices (at most 40 pig owners per village). All questionnaires were conducted and recorded on Personal Digital Assistants (PDAs). The PDAs recorded the geographical coordinates of each concession.

### Wealth indicator

We used indicators of wealth as suggested in Gwatkin *et al*. (2000) [18] to estimate the wealth quintile of each household. Several wealth indicators were missing and therefore imputed using the missMDA package in R [19,20]. Principle Component Analysis was run in R using the PCA command of the FactoMineR package [21]. The imputed and known were exported back to Stata where PCA analysis was conducted and the fitted values were used to obtain the percentiles that were used to classify the households into 10 wealth groups.

### Soil sampling

Between 18 March and 25 November 2014, the field team returned to each village to take soil samples to measure soil composition and pH using the LaMotte Soil Texture Unit test (code 1067) and LaMotte pH Test kit (code 5024), respectively. The soil composition test estimates the percentage of sand, silt and clay in the soil. In each village, a soil sample was taken in each of the four cardinal directions (within 2 km) using a plastic pot filled to 125 ml, having scraped the soil surface to a depth of about 5 to 10 cm. The four samples were then thoroughly mixed, dried and cleaned of coarse elements such as stones. A subsample of this mixture (about 125 ml) was then taken and stored for analysis of soil composition and pH, according to LaMotte procedure.

### Blood sampling

After an average of 7 weeks (range of 0 to 140 days), a physician went to each village to take blood samples from the 60 participants who consented to the serological component of the study and to examine all participants who had screened positive for seizures, epilepsy or severe chronic headache. Any participant confirmed as having epileptic seizures, epilepsy or severe chronic headache who had not initially consented to the serological follow-up were asked to provide a blood sample for (clinical) diagnosis purposes. However, to avoid an over-selection of people with neurological symptoms in our analysis, only those who initially consented to the serological analysis component of the study are included in the analysis. Blood samples of 8 ml were drawn by venipuncture using a syringe, preferably at the antebrachium vein, using 10 ml Venosafe serum gel tubes. The tubes were placed in a cooler, left to decant, and the sera were collected and put in two pre-labelled tubes at the end of each day or the following day. The sera were placed in freezers (-20°C) at most three days after the blood draw. The sera were brought to the IRSS (*Institut de Recherche en Sciences de la Santé*) in Bobo-Dioulasso every 4 to 8 weeks and kept thereon in a freezer at -20°C until analyses took place.

### Serological test

The serum samples were tested for presence of excretory secretory circulating antigens of the metacestode of *T*. *solium* using the B158/B60 enzyme-linked immunosorbent assay (ELISA) [13]. The test was found to have a sensitivity of 90% (95%BCI: 80–99%) and a specificity of 98% (95%BCI: 90–99%) to detect current infection in a study conducted in Ecuador [22].

### Statistical analyses

Descriptive analyses on the study population were first conducted, followed by assessing the association between each potential risk factor and the prevalence of current cysticercus infection at the individual-level and at the village-level. Household characteristics measured through the mother and chief questionnaires were attributed to each individual since only one individual was sampled per household (and concession). Consequently household-level variables were included at the individual-level in all analyses. All descriptive analyses were conducted in Stata 13.1.

Data obtained from pig owners were considered as village-level variables. These included the percentage of pig owners letting their animals roam all seasons, practicing home slaughtering of pigs and asking for inspection at the time of slaughter. Soil composition, soil pH and the season when human samples were collected (dry vs wet) were also considered as village-level variables. The effect of the coverage of the 2012 filiariasis mass drug delivery campaign, which provided albendazole (400 mg) with ivermectin, obtained from the Ministry of Health, was also explored at the village-level.

The effects of variables which may impact the contamination of the environment with human feces were explored at the individual-level and at the village-level. These included the use of latrines to defecate reported by interviewees, the access to a latrine as reported by the mother, the household wealth quintile (as an indicator of general hygiene and sanitation), the knowledge about taeniasis including report of self-infection, and the reporting of pork consumption at home and outside the home.

Individual-level variables explored that were not likely to directly influence environmental contamination included age, gender, education and occupation, although the effect of age was modelled separately for each province. Age was categorized because it was not linear in the logits.

The only concession-level variable explored was the type of concession sampled (i.e. sow, piglet or other).

Given the sampling strategy, concession, household and individual characteristics were all attributed to the individual-level in the analysis. To take the stratified nature of the sampling into account, models including the type of concession sampled were run but did not modify the estimated medians and 95%BCI of the estimates. Hence, results from the simpler model are being reported here.

Bayesian hierarchical logistic models were fitted to estimate the prevalence odds ratios between each variable of interest and the prevalence of current cysticercus infection. Some models were also run with Bayesian hierarchical log-binomial models and resulted in similar estimates when convergence was achieved. At the first level, current cysticercus infection was assumed to follow a Bernoulli distribution. The logit of this distribution was modeled using the individual-level variables and village-level random-effect intercepts. At the second level, each village-level intercept was assumed to follow a normal distribution. The mean of the random-effect intercepts were modelled as a linear regression using the village-level variables, including the province in some models. The effect of the provinces with a random-effect on villages was not important, and therefore all presented models exclude province effects. Diffuse priors were used for all regression coefficients. Missing independent variable values were imputed using the mean value of non-missing data, assuming an ignorable missingness mechanism. When the mean values varied by province, province-level means were used for imputation. Fit was measured by comparing deviances. Convergence was assessed by looking at the history and b Rubin Gelman plots. Some of the more complex models required large numbers of iterations and thinning of 100 to obtain stable estimates. All models were run in WinBUGS [23].

### Ethical considerations

The protocol and consent forms were approved by the University of Oklahoma Health Sciences Center Institutional Review Board and by the Centre MURAZ ethical review panel (Burkina Faso). All participants were read the consent form and any questions they had were answered to the best knowledge of the field staff. Each participant was given a bar of soap to thank them for their time. Information about the study provided in the consent forms were read and explained to each potential participant (mother of the household, chief of the household, participant, pig owners) by the field workers who took the time to answer all questions. Consenting participants signed the consent forms when able or put a cross when not. All consents were witnessed by a local villager. Children aged more than 10 were asked for their assent, but parents consented for all children aged less than 18 years old.

## Results

### Study population

A total of 4795 villagers consented to being screened for epilepsy and severe chronic headaches three times during the course of the parent community-based randomized trial. All three mother, chief of the household and individual questionnaires were missing for three individuals who were excluded from the analyses. Of the remaining 4792 participants, 4788, 4772 and 4778 had information from the chief questionnaire, mother questionnaire or individual questionnaire available, respectively. A total of 3609 participants consented to participate in the serological component of the parent randomized trial and provided sufficient blood at baseline to be analyzed. The characteristics of individuals participating in the serological and screening-only component of the study are described in Table 1. Since individuals living in concessions where pigs were being raised were first asked to participate in the serological component of the study, there was a larger proportion of participants who provided blood who either raised pigs or consumed pork. The participation proportion was similar according to other characteristics although females and more educated people tended to be more likely to consent to the serological follow-up component of the study.

**Table 1:** Comparison of socio-demographic characteristics of 4792 individuals who consented to the serological (n = 3609) and screening- only (n = 1183) component of a study conducted in 60 villages of Burkina Faso in 2011–2012.

		Seroconsent		
Variable	Categories	No	Yes	Difference
Province	Sanguié	386	1209 (75.8%)	
	Nayala	202	598 (74.7%)	-1.0% (-4.7%; 2.6%)
	Boulkiemdé	595	1805 (75.2%)	-0.5% (-3.3%; 2.1%)
Age (35 missing)	> 18	621	1844 (74.8%)	
	18 - ≤ 49	291	1016 (77.8%)	2.9% (0.01%; 5.8%)
	50+	261	724 (73.5%)	-1.3% (-4.6; 1.9%)
Gender (15 missing)	Females	594	1975 (76.9%)	
	Males	588	1620 (73.4%)	-3.5% (-6.0%;-1.1%)
School attendance (17 missing)	No	868	2522 (74.4%)	
	Yes	314	1071 (77.3%)	2.9% (0.3%; 5.6%)
Ever had pigs (18 missing)	No	848	2377 (73.7%)	
	Yes	334	1215 (78.4%)	4.7% (2.2%; 7.3%)
Eating pork now (20 missing)	No	462	1154 (71.4%)	
	Yes	719	2437 (77.2%)	5.8% (3.2%-8.5%)
Pork eating history (18 missing)	Never	355	868 (71.0%)	
	Now	719	2437 (77.2%)	6.2% (3.3%;9.2%)
	In the past	108	287 (72.7%)	1.7% (-3.4%; 6.8%)
Concession type	Sow	107	495 (82.2%)	
	Piglet	393	1362 (77.6%)	-4.6% (-8.2%; -1.0%)
	Any	682	1755 (72.0%)	-10.2% (-13.7%; -6.7%)
HH owns pigs (3 missing)	No	398	1001 (71.6%)	
	Yes	784	2606 (76.9%)	5.3% (2.6%; 8.1%)
Where pork is eaten (19 missing)	At home	372	1230 (76.8%)	
	Other concession	106	424 (80.0%)	3.2% (-1.0%; 7.2%)
	Village market	172	577 (77.0%)	2.6% (-3.4%; 3.9%)
	Other village market	69	206 (74.9%)	-1.9% (-7.4%; 3.7%)
Ever heard of pig cysts (among 1549 ever had pigs)	No	282	1043 (78.7%)	
	Yes	52	172 (76.8%)	-1.9% (-7.9%; 4.0%)
**Use toilet to defecate (18 missing)**	No	1065	3124 (74.6%)	
	Yes	117	468 (80.0%)	5.4% (1.9%; 8.9%)
Access to a latrine (reported by mother, 50 missing)	No	1050	3132 (74.9%)	
	Yes	121	439 (78.4%)	3.5% (-0.2%; 7.2%)
HH has a latrine (reported by chief, 4 missing)	No	1041	3118 (75.0%)	
	Yes	140	489 (77.7%)	2.7% (-0.7%; 6.3%)
Heard about tapeworm (18 missing)	No	437	1342 (75.4%)	
	Yes, had it	94	353 (79.0%)	3.5% (-0.7%; 7.8%)
	Yes, did not have it	651	1897 (74.5%)	-1.0% (-2.2%; 4.7%)
Wealth quintile (1 missing)	0	261	697 (72.8%)	
	1	231	727 (75.9%)	3.1% (-0.8%; 7.0%)
	2	233	726 (75.7%)	2.9% (-1.0%; 6.9%)
	3	235	725 (75.5%)	2.8% (-1.2%; 6.7%)
	4	222	734 (76.8%)	4.0% (0.1%; 7.9%)
**Occupation (17 missing)**	Student/pupil	235	789 (77.1%)	
	Farmer	491	1332 (73.1%)	-4.0% (-7.3%; -0.7%)
	Housewife / cleaner	392	1283 (76.6%)	-0.5% (-3.7%; 2.8%)
	Salaried / commerce / unemployed	64	189 (74.7%)	-2.3% (-8.3%; 3.6%)

### Prevalence of current cysticercus infection

**Fig 3:**
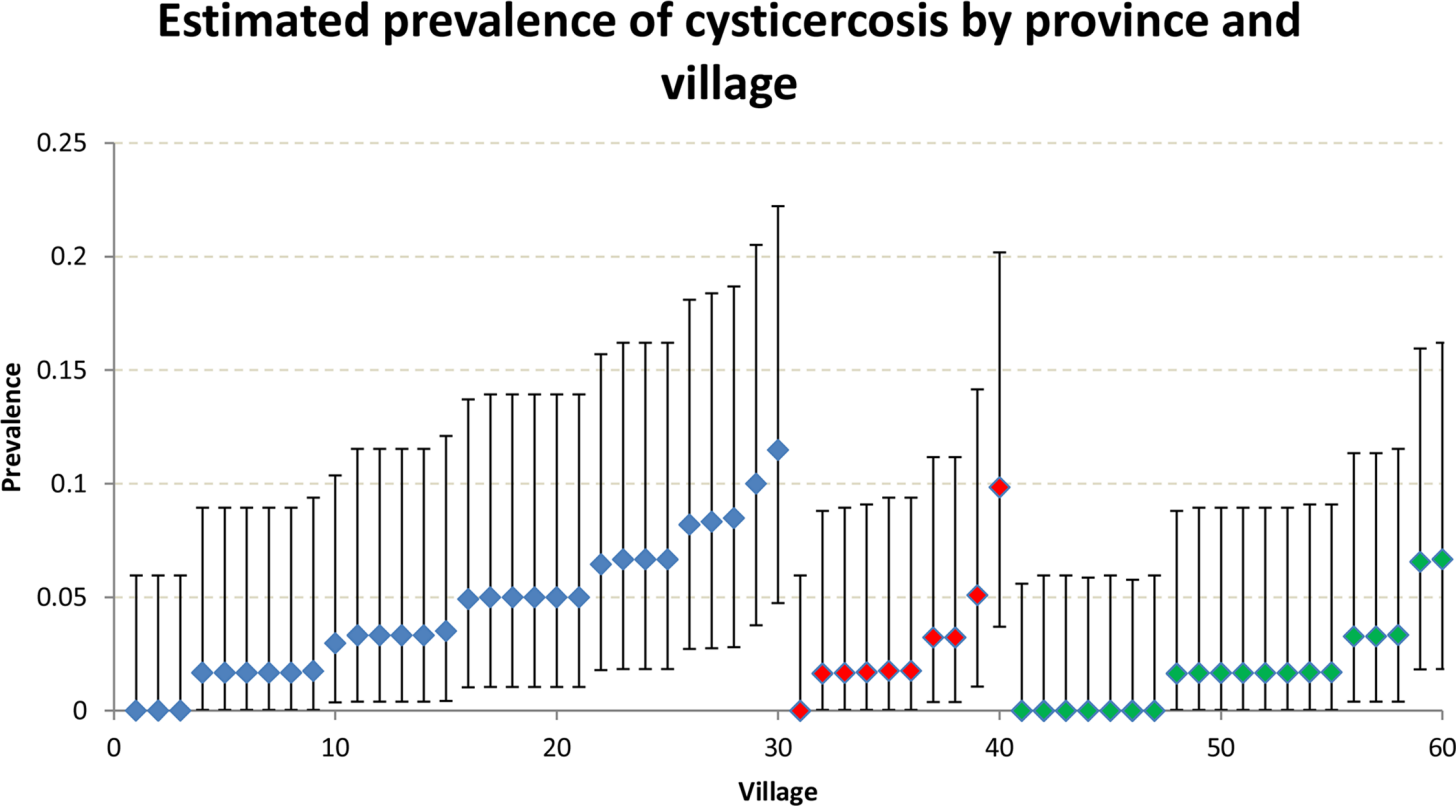
Village-level seroprevalence of current infection with cysticercosis among 3609 individuals living in 60 villages of Burkina Faso, 2011–2012. Blue diamonds: Boulkiemdé, red diamonds: Nayala; green diamonds: Sanguié

A total of 120 individuals tested positive for current infection with cysticerci. The prevalence of current cysticercosis varied considerably across provinces and villages (Figs 1 and 3) ranging from 0% to 11.5%, although the 95%CI were wide. In the Province of Sanguié, no individual tested positive in seven (35%) of the 20 villages studied. In contrast, this was the case in only three (10%) and one (10%) villages in Boulkiemdé and Nayala, respectively.

### Factors associated with the prevalence of infection in univariate analyses

Table 2 provides estimates of the prevalence according to different individual-level characteristics of the participants as well as their associated prevalence proportion ratios. The univariate analyses suggested that older males, people living in a household with lower wealth quintiles and those consuming pork had higher prevalences of infection. In addition, access to a latrine as reported by the mother of the household was associated with a reduced prevalence of infection.

**Table 2:** Univariate association between potential individual-level risk factors and the prevalence of current infection with cysticercosis among 3609 individuals providing serum in 60 villages of Burkina Faso, 2011–2012.

		Sero-status		Prevalence ratio (95% CI)
Variable	Categories	Positive	Negative	
Province	Sanguié	22 (1.8%)	1185	Reference
	Nayala	18 (3.0%)	580	1.65 (0.89; 3.06)
	Boulkiemdé	80 (4.4%)	1724	2.43 (1.53; 3.88)
Age in Boulkiemdé (11 missing)	> 18	15 (1.7%)	871	Reference
	18 - ≤ 49	24 (4.9%)	467	2.89 (1.53; 5.45)
	50+	41 (9.9%)	375	5.82 (3.26; 10.40)
Age in Nayala (1 missing)	> 18	6 (2.3%)	250	Reference
	18 - ≤ 49	8 (3.8%)	201	1.63 (0.58; 4.63)
	50+	4 (3.0%)	128	1.29 (0.37; 4.50)
Age in Sanguié (13 missing)	> 18	5 (0.7%)	697	Reference
	18 - ≤ 49	10 (3.2%)	306	4.44 (1.53; 12.89)
	50+	6 (3.4%)	170	4.79 (1.48; 15.50)
Gender (15 missing)	Females	39 (2.0%)	1936	Reference
	Males	81 (5.0%)	1539	2.53 (1.74; 3.69)
School attendance (16 missing)	No	92 (3.7%)	2430	Reference
	Yes	28 (2.6%)	1043	0.72 (0.47; 1.09)
Ever had pigs (17 missing)	No	81 (3.41%)	2296	Reference
	Yes	39 (3.21%)	1176	0.94 (0.65; 1.37)
Eating pork now (18 missing)	No	23 (2.0%	1131	Reference
	Yes	97 (4.0%)	2340	1.99 (1.27; 3.13)
Pork eating history (17 missing)	Never	11 (1.3%)	857	Reference
	Now	97 (4.0%)	2340	3.14 (1.69; 5.83)
	In the past	12 (4.2%)	275	3.30 (1.47; 7.40)
Sampling concession type (0 missing)	Sow	23 (4.7%)	472	1.63 (1.00; 2.64)
	Piglet	47 (3.5%)	1313	1.21 (0.82; 1.79)
	Any	50 (2.9%)	1704	Reference
HH owns pigs (3 missing)	No	18 (1.8%)	983	Reference
	Yes	102(3.9%)	2504	2.18 (1.33; 3.57)
Where pork is eaten (19 missing)	Never eats pork	11 (1.3%)	856	Reference
	At home only	43 (3.5%)	1187	2.76 (1.42–5.31)
	Other concession	6 (1.4%)	418	1.11 (0.42–3.00)
	Village market	30 (5.2%)	547	4.10 (2.07–8.11)
	Other village market	18 (8.7%)	188	6.89 (3.30–14.36)
	Ate pork before, not anymore	12 (4.2%)	275	3.30 (1.47–7.39)
Ever heard of pig cysts (17 missing)	No	101 (4.4%)	2190	3.02 (1.86–4.90)
	Yes	19 (1.5%)	1282	Reference
Use toilet to defecate (17 missing)	No	111 (3.6%)	3013	0.54 (0.28; 1.06)
	Yes	9 (1.9%)	459	Reference
Access to a latrine (reported by mother) (37 missing)	No	112 (3.6%)	3020	Reference
	Yes	7 (1.6%)	432	0.45 (0.21; 0.95)
HH has a latrine (reported by chief, 2 missing)	No	111 (3.6%)	3007	Reference
	Yes	9 (1.9%)	480	0.52 (0.26; 1.01)
Ever heard about tapeworm (18 missing)	No	23 (1.7%)	1319	Reference
	Yes, had it	21 (6.0%)	332	3.47 (1.94–6.20)
	Yes, but did not have it	76 (4.0%)	1821	2.34 (1.47–3.71)
Wealth quintile	1	28 (4.0%)	669	1.47 (0.84–2.59)
	2	27 (3.7%)	700	1.36 (0.77–2.41)
	3	27 (3.7%)	699	1.36 (0.77–2.41)
	4	18 (2.5%)	707	0.91 (0.49–1.71)
	5	20 (2.7%)	714	Reference
Occupation (17 missing)	Student/pupil	12 (1.7%)	704	Reference
	Farmer	71 (5.3%)	1261	3.18 (1.73–5.82)
	Housewife / cleaner	31 (2.4%)	1283	1.44 (0.75–2.79)
	Salaried / commerce / unemployed	6 (2.3%)	256	1.37 (0.52–3.60)

Table 3 shows the linear regression coefficients between the prevalence of infection in each village and the village-level variables. The percentage of participants reporting eating pork, and particularly those eating pork in someone’s or their household, and the percentage of participants reporting having had a tapeworm infection were associated with a slight increase in the village-level prevalence of infection. The percentages of silt in the soil and of sand in the soil were associated with a decrease and increase in the prevalence, respectively.

**Table 3:** Correlations between the village-level prevalence of current infection with cysticercosis and village-level variables in 60 villages of Burkina Faso, 2011–2012.

Variable	Linear regression correlation coefficient (95% CI)
Percentage of pigs roaming or tethered during the rainy season and roaming during the dry season	-0.01 (-0.06;0.04)
Percentage of households practicing home slaughtering	0.02 (-0.05;0.09)
Percentage of household with home slaughtering for which meat inspection is practiced	-0.01 (-0.06;0.05)
Percentage of households owning pigs	0.04 (0.00;0.08)
pH level in soil	-0.86 (-1.97;0.25)
Percentage of silt in soil	-0.07 (-0.13;-0.01)
Percentage of sand in soil	0.06 (0.00;0.11)
Percentage of clay in soil	-0.01 (-0.11;0.10)
Percentage self-reporting using latrines to defecate	-0.01 (-0.07;0.05)
Percentage of households in which mothers declared that family members had access to a latrine	0.00 (-0.05;0.06)
Percentage with wealth quintile of 4 or 5	0.03 (-0.03;0.09)
Percentage of participants who reported ever having had a tapeworm	0.14 (0.00;0.28)
Percentage of participants who reported ever heard about tapeworm, but never had one	-0.03 (-0.11;0.05)
Percentage of participants declaring eating pork	0.04 (0.00;0.08)
Percentage of participants declaring eating pork only at someone’s household (including own)	0.08 (0.02;0.13)
Percentage of participants declaring eating pork at the market (village market or other)	0.02 (-0.06;0,10)

### Factors associated with the prevalence of infection in multivariable analyses

Table 4 shows the results of three candidate models with the lowest deviances. Models 2 and 3 include soil indicators which were measured up to 36 months after the baseline visit while model 1 does not include these variables. The individual-level variables were common to all models and all resulted in similar magnitudes of association with the prevalence odds of infection. Being aged more than 50 years old had a stronger effect on the prevalence odds in Boulkiemdé than in the other two provinces. Males had cysticercosis prevalence odds of nearly 2.6 when compared to females. Eating pork at another village market had the strongest effect, while eating pork at the village market or at home also increased the prevalence odds of infection when compared to never having eaten pork. Living in a household with higher wealth quintiles and having access to a latrine both decreased the prevalence odds of infection, with those not having access to a latrine having a prevalence odds of about 2.8 times higher than those having access to a latrine. The effect of a self-history of taeniosis and knowledge of taeniosis became negligible in models adjusted for age (ie age was a strong confounder of taeniosis history and knowledge).

**Table 4:** Prevalence odds ratios (95% Bayesian Credible Interval) of individual and village-level variables associated with the prevalence of current infection in 60 villages of Burkina Faso (2011–2012) from three hierarchical Bayesian hierarchical logistic models.

Variable	Exposure	Reference	POR (95%BCI)		
			Model 1	Model 2	Model 3
**Individual-level variables**					
Age in Boulkiemdé	31–50 years old	6–30 years old	3.84 (2.11;7.11)	3.43 (1.89;6.26)	3.59 (1.95;6.58)
	More than 50 years old		7.32 (4.25;12.85)	6.53 (3.82;11.37)	6.73 (3.91;11.85)
Age in Nayala	31–50 years old	6–30 years old	2.92 (1.14;6.75)	3.18 (1.24;7.32)	3.09 (1.20;7.17)
	More than 50 years old		1.91 (0.51;5.49)	2.02 (0.56;5.77)	1.97 (0.53;5.65)
Age in Sanguié	31–50 years old	6–30 years old	2.21 (0.77;5.44)	2.43 (1.05;5.32)	2.27 (0.98;4.93)
	More than 50 years old		2.06 (0.90;4.44)	2.59 (0.90;6.42)	2.42 (0.84;5.99)
Gender	Male	Female	2.58 (1.69;4.01)	2.60 (1.71;4.03)	2.58 (1.69;4.01)
Pork consumption	Eats pork at home only	Never ate pork	3.31 (1.73;6.95)	3.41 (1.74;7.15)	3.44 (1.77;7.25)
	Eats pork in other home		1.52 (0.50;4.19)	1.57 (0.51;4.35)	1.63 (0.53;4.54)
	Eats pork at the village market		3.11 (1.54;6.73)	3.17 (1.56;6.79)	3.25 (1.60;7.06)
	Eats pork at another village market		4.78 (2.15;11.26)	5.24 (2.32;12.22)	5.18 (2.29;12.22)
	Ate pork before, not anymore		2.07 (0.86;5.01)	2.10 (0.87;5.01)	2.11 (0.87;5.10)
Wealth quintile	4–5	1-2-3	0.64 (0.42;0.95)	0.61 (0.40;0.91)	0.63 (0.41;0.94)
Household members have access to a latrine	Yes	No	0.38 (0.15;0.80)	0.36 (0.15;0.78)	0.37 (0.15;0.80)
**Village-level variables**					
Percentage of pigs not penned during the rainy season	Per percent increase		2.48 (0.88;7.00)	2.74 (1.02;7.43)	2.80 (1.01;8.72)
pH of the soil	Per unit increase in pH			0.69 (0.50; 0.93)	
Percentage of sand in the soil	Per percent increase			1.02 (1.00;1.04)	1.02 (1.00;1.04)

The major differences in the three models come from the inclusion of the type of soil and soil pH. When these variables are excluded, the percentage of pigs not penned during the rainy season (i.e. left roaming or tethered) led to a weaker association with the prevalence odds of cysticercosis. An increase in the alkalinity of the soil was associated with a lower prevalence odds of cysticercosis. This effect was only noted when the percentage of silt or sand in the soil was also included in the model. We present here the effect of the percentage of sand, but the percentage of silt had an opposite effect of similar magnitude to that of sand.

## Discussion

This cross-sectional study is the most widespread ever conducted, estimating factors associated with the prevalence of current infection of human cysticercosis. Our study is unique in its inclusion of 60 villages located in three provinces and the evaluation of over 3600 people living in these villages. The inclusion of only one individual per household and concession reduced the dependence among observations, thus maximizing the power to detect individual and household-level factors associated with the prevalence. Our hierarchical model also includes potential risk factors measured at the individual- and village-level, thus respecting the sample size of each unit. Finally, by including the village as random-effects, and having explored the impact of incorporating the type of concession which was part of the sampling scheme, we are effectively using a model-dependent approach to adjusting for the sampling scheme, thus reducing the potential biases which may be introduced by clustered sampling [24]. To our knowledge, only two studies conducted in Sub-Saharan Africa had adjusted for the cluster nature of the sampling or the infection [7,25].

We found that the prevalence of current infection with cysticercosis varied from 0% to 11.5% in the sampled 60 villages. These villages were sampled with the goal of conducting a community-based randomized controlled trial and participants were selected based on the presence of pig raising in their household. Therefore, the overall prevalence cannot be generalized to the three provinces nor to the country as a whole. Nonetheless, the village-level prevalences of current infection with cysticerci measured with the AgELISA are within the range of those reported by others using the same test in community-based studies conducted in three rural communities of West Cameroon (from 0.4% (0.2%;0.7%) to 3% (0.3%;11.2%) depending on the locality) [7], one small village in Sénégal (with 7.7% (5.3%-10.7%)) [8], and 20 villages in Zambia (with 5.8% (4.1%-7.5%) [26]. Other community-based studies have reported higher overall prevalences of current cysticercus infection (ie not village-specific) in one village in the Democratic Republic of Congo (with 21.6% (18.2%-25.0%) [25] and 13 villages in Tanzania where very high porcine cysticercosis prevalence levels had been reported (with 16.7% (14.2%;19.2%)) [27].

A unique characteristic of our study is its ability to explore between-villages variation in prevalence. Although the villages were sampled with a set of inclusion criteria necessary for the randomized trial, considerable variation in the prevalence was observed among them as well as among the three sampled provinces, although the credible intervals were wide. Such variation in areas was also observed in a study conducted in three areas of West Cameroon [7], different districts of a village in the Democratic Republic of Congo [25], and six departments of Bénin [9], although the latter study used an EITB to detect exposure to infection [12] instead of current infection. This confirms observations by others that current cysticercus infections in humans occur in clusters [26, 28–30], often around taeniosis carriers. The very clustered nature of cysticercosis calls for great care in attempting to generalize results from studies conducted in a small number of villages or communities to larger areas or to a country.

The individual-level factors found to be associated with the prevalence odds of current infection are similar to those reported in other community-based studies conducted in Sub-Saharan Africa. An increased prevalence odds of current cysticercus infection in adults aged 30 or more as compared to individuals aged 7–30 years old was observed in all three provinces. This confirms what was observed in a study of 720 participants living in 20 villages of Zambia where the prevalence odds increased after the age of 30 years old when compared to those aged 0–9 years old [26] and a study in Tanzania where the prevalence odds was increased in individuals aged 36 or more when compared to those aged 15–25 years old [27]. Moreover, in the province of Boulkiemdé, a further increase in the prevalence odds was observed in people aged 50 years old or more. Such increase in prevalence in older people has been observed in studies conducted in the Democratic Republic of Congo (POR of 2.8 95%CI: 1.14; 3.81 for those aged 70 or more compared to those aged 0–9 years old) [25] and in West Cameroon (seroprevalence of 2% in those aged 46 years old or more and 0.1% in those aged 15 or less) [7]. A study conducted in 1989 in Bénin found an age-pattern of sero-prevalence of exposure to cysticercosis which is very similar to that observed in Boulkiemdé, with an initial increase at 30 years of age, followed by a further increase after 50 years old [9]. The Zambian study did seem to show a tendency for higher prevalence of current cysticercosis after the age of 50, but the small sample size in older age groups may have reduced the power to detect such increase [26]. A study conducted in Ecuador suggested that the increase in current infection in older age could be linked to reduced immunity in older age groups [31]. This is further supported by findings from a cohort study conducted in Zambia which showed that while sero-reversion rates were higher than seroconversion rates in people aged less than 60, such difference disappeared in people aged 60 years old or more [32]. The reason why an additional increase in older ages was only observed in one province is difficult to explain and may require future studies, but may be linked to differential at risk behavior in this group of older men not captured elsewhere.

The increase of prevalence odds of current infection with or exposure to cysticercosis in males has been reported by some community-based studies conducted in Sub-Saharan Africa [9,15,25,33], but not all [7,8,26]. The study conducted in the Mbozi district of Tanzania reported an impact of gender on exposure to cysticercosis measured with the EITB, but the reporting is problematic because the gender among whom the prevalence odds is reported to be increased is inconsistent (males in the results section and females in the discussion section and Table 3) [27]. In that same study, the effect of washing hands by dipping in multiusers buckets was associated with a decrease prevalence odds of exposure to infection and an increased prevalence odds of current infection, casting doubts about the reported results. The difference in results between studies could be real, or could be attributable to the reporting of crude associations in some studies [9] and that resulting from multivariable analyses in others [7,8,15,25, 26,33]. In our study, the association between being male and the prevalence odds persisted after adjusting for pork consumption, wealth quintile of the household, and access to a latrine. This confirms that factors which bring males to get exposed to *T*. *solium* eggs more than females, such as poor hand hygiene or consumption of fresh produce such as fruits and vegetable that have not been cleaned properly, or eating meals outside the home that could be prepared by foodhandlers infected with taeniasis may be playing a role. Behavioral studies comparing male and female food consumption and hand hygiene behavior are warranted to develop future interventions.

Where pork is consumed was found to have an impact on current infection with cysticerci. Since cysticercosis can be acquired by either self-infection or through food or hands contaminated with the eggs of *T*. *solium*, and since the effect of a self-reported history of taeniosis became negligible once gender and age were included in the model, contamination of food with *T*. *solium* eggs may play an important role in this population. To our knowledge, this is the first time that the effect of where the pork is consumed is reported. In Sub-Saharan Africa, only one study in Zambia found that not boiling pork before its consumption played a role among older females based on a classification tree model [33]. Other studies conducted in Latin America, although in univariate analyses, had reported an association between pork consumption and exposure to cysticercosis [34,35].

Having access to a latrine was associated with reduced prevalence odds of current infection with cysticerci. This had been mentioned in the small scale study of Secka *et al*. conducted in Sénégal [8], but was not confirmed in a multivariable analysis. It was also observed in relation to the prevalence of exposure to infection in univariate analyses in Colombia [35,36] and Honduras [37]. The effect of having access to a latrine by family members had a stronger effect than that of participants declaring that they used a latrine to defecate. This is in agreement with a recent qualitative research conducted in Eastern Zambia which showed that latrines are usually considered as public among neighbors [38]. Our results support that access to latrines is an indicator of environmental contamination with taeniid eggs around the household.

Poor living conditions, as indicated by the three lowest wealth quintiles in our study, have also been reported to be associated with increased prevalence odds in previous studies, in these cases using univariate analyses. In an urban area of Honduras, Sanchez *et al*. [37] reported that several indicators of poor living conditions such as raising pigs, lack of potable water, lack of sanitary toilets and earthen floor were associated with an increased prevalence odds of exposure to cysticercosis. Poor hygienic conditions of the household was associated with an increased prevalence odds of exposure to infection in the area of Morelos, Mexico [34].

The inclusion of 60 villages in our study allowed us to assess the village-level effect of the type of pig management on the prevalence odds of current cysticercus infection. To our knowledge, this is the first time that a study has a number of sampled villages large enough to explore and measure village-level factors. Previous studies conducted in Latin America had found that ownership of pigs was associated with the prevalence odds of exposure to infection [28,35–37]; we found that how pigs are managed at the village-level has an impact. No previous studies conducted in Sub-Saharan Africa had found such effect. Indeed, ownership of pigs by the respondent did not yield an association with the prevalence of cysticercosis while pig ownership at the household level did in univariate analyses. This should be considered in future studies. In the study areas, almost all pigs were left to roam during the dry season. However, villages where a larger percentage of pigs were not penned during the rainy season had higher prevalence odds of current cysticercosis.

This design also allowed us to find an association between the type of soil and the soil pH and the prevalence odds of current infection. An increase in the pH of the soil was associated with lower village-level prevalence odds of current infection. In an experimental study of inactivation of *T*. *solium* eggs in different temperature, dryness and pH conditions, an increase in alkalinity in an alkaline environment (pH from 12.1 to 12.7) was linked to an increase in inactivation rates of the eggs while the opposite was true for in an acidic environment (pH from 5.1 to 5.5) [39]. The soil pH in our study villages was at an average of 6.8 with a range from 5.4 to 8.2. The soil was therefore nearly neutral on average, and it is difficult to say if the results are consistent to these found in [39]. However, it could be hypothesize that the eggs are more tolerant to the generally more acidic environment of the gastro-intestinal tract, which could favor better egg survival in slightly more acidic soil. An increase in the percentage of sand in the soil was associated with an increased village-level prevalence odds of current infection. Perhaps taeniid eggs are more easily disseminated from sandy soil onto vegetables and water through wind. The fact that the soil was sampled nearly three years after the baseline study may also have an impact, although it is unlikely for the soil to change its pH extensively through time.

A self-report of a history of infection with taeniosis was associated with current infection with cysticerci only in univariate analyses. This association was confounded by age, and became non-significant in the hierarchical model also including pork management variable. Others had found associations between the self-report of taeniosis and exposure to cysticercosis in univariate analyses [34]. This underlines the importance of conducting multivariate analyses to identify factors with the strongest impact on infection.

This study had some limitations. The most important one is that the soil samples were collected nearly three years after the baseline study, therefore, these associations should be interpreted with great care. This is why results were presented with and without including the soil analyses. Future studies using follow-up data of the randomized trial among the control group should be able to confirm (or not) this association. Only one person per household was sampled and therefore, factors affecting clusters within households could not be evaluated.

In conclusion, this study is the first to assess the association between several individual-, household, and village-level variables and the prevalence odds of cysticercosis in humans at a large scale. We found that factors linked to people, pigs, and the environment were of importance. This further supports the need for a One Health approach to control this infection.

## Supporting Information

S1 Checklist STROBE checklist. (DOC)

S1 Dataset Table ind_lev with individual- and household-level variables villages, province, gender, access to latrine, where pork is eaten, wealth quintiles and age category. Table vill_level with with percent of roaming pigs, soil pH level and percentage of sand in the soil by village.
(XLS)

S1 Fig A pig searching for food near a waste area in one of the study villages. (JPG)
