# Supplementary material for: Prevalence of and Factors Associated with Human Cysticercosis in 60 Villages in Three Provinces of Burkina Faso
Source: PLoS Negl Trop Dis. 2015 Nov 20;9(11):e0004248. doi: 10.1371/journal.pntd.0004248 (PMC4654529; doi:10.1371/journal.pntd.0004248)
Supplement: S1 Checklist — (DOC) [file pntd.0004248.s001.doc]

STROBE Statement—checklist of items that should be included in reports of observational studies

|  | Item No | Recommendation | Page No. | Relevant text from manuscript |
| --- | --- | --- | --- | --- |
| **Title and abstract** | 1 | *(*a) Indicate the study’s design with a commonly used term in the title or the abstract | 2 | Mentioned in abstract |
|  |  | (*b*) Provide in the abstract an informative and balanced summary of what was done and what was found | 2 | Done |
| Introduction | | |  |  |
| Background/rationale | 2 | Explain the scientific background and rationale for the investigation being reported | 4-5 | Done |
| Objectives | 3 | State specific objectives, including any prespecified hypotheses | 5 | Main objective mentioned in last paragraph of introduction. |
| Methods | | |  |  |
| Study design | 4 | Present key elements of study design early in the paper | 5 | Mentioned in “Study design” section. |
| Setting | 5 | Describe the setting, locations, and relevant dates, including periods of recruitment, exposure, follow-up, and data collection | 5-6 | Mentioned in “Study area and selection of study villages” |
| Participants | 6 | (*a*) *Cohort study*—Give the eligibility criteria, and the sources and methods of selection of participants. Describe methods of follow-up  *Case-control study*—Give the eligibility criteria, and the sources and methods of case ascertainment and control selection. Give the rationale for the choice of cases and controls  *Cross-sectional study*—Give the eligibility criteria, and the sources and methods of selection of participants | 5-9 | NA  NA  Described in details in these pages |
|  |  | (*b*) *Cohort study*—For matched studies, give matching criteria and number of exposed and unexposed  *Case-control study*—For matched studies, give matching criteria and the number of controls per case | NA | NA  NA |
| Variables | 7 | Clearly define all outcomes, exposures, predictors, potential confounders, and effect modifiers. Give diagnostic criteria, if applicable | 9-12 | Described in “Questionnaires”, “Wealth Indicator”, “Soil sampling”, “Blood sampling” and “Serological test” sections |
| Data sources/ measurement | 8* | For each variable of interest, give sources of data and details of methods of assessment (measurement). Describe comparability of assessment methods if there is more than one group | 9-12 | See Item (7) |
| Bias | 9 | Describe any efforts to address potential sources of bias | 9-12 | Previously used methods were applied to measure variables of interest. Multivariable analyses were used to measure confounding. Village-level clustering included to adjust for non-independence. |
| Study size | 10 | Explain how the study size was arrived at | 9 | Study size was based on the effectiveness of the randomized community trial based on expected follow-up data. |
| Quantitative variables | 11 | Explain how quantitative variables were handled in the analyses. If applicable, describe which groupings were chosen and why | 12-13 | All variables included, except for wealth quintiles and age, were categorical. Age categorization was based on the distribution of the log odds. |
| Statistical methods | 12 | *(*a) Describe all statistical methods, including those used to control for confounding | 12-14 | Done |
|  |  | (*b*) Describe any methods used to examine subgroups and interactions | 13 and results | All models and subgroup analyses examined were described in the methods, under statistical analyses. |
|  |  | (*c*) Explain how missing data were addressed | 14 and results | Description of how missing data were addressed are described in the statistical analyses section and in the first paragraph of the results. |
|  |  | (*d*) *Cohort study*—If applicable, explain how loss to follow-up was addressed  *Case-control study*—If applicable, explain how matching of cases and controls was addressed  *Cross-sectional study*—If applicable, describe analytical methods taking account of sampling strategy | 13 | As described in the methods, models were run to take into account the sampling strategy. |
|  |  | (*e*) Describe any sensitivity analyses | 13 | The statistical analyses section describes that two models were run to examine the association between the variables of interest and the outcome. |

Continued on next page

| Results | | | Page No. | Relevant text from manuscript |
| --- | --- | --- | --- | --- |
| Participants | 13* | (a) Report numbers of individuals at each stage of study—eg numbers potentially eligible, examined for eligibility, confirmed eligible, included in the study, completing follow-up, and analysed | 15 | Done |
|  |  | (b) Give reasons for non-participation at each stage |  | There was minimal refusal to participate |
|  |  | (c) Consider use of a flow diagram | Figure 2 | Flow diagram is presented for the sampling procedure of the concessions. |
| Descriptive data | 14* | (a) Give characteristics of study participants (eg demographic, clinical, social) and information on exposures and potential confounders | 15-16, Table 1 | Done |
|  |  | (b) Indicate number of participants with missing data for each variable of interest | 15 | Done |
|  |  | (c) *Cohort study*—Summarise follow-up time (eg, average and total amount) | NA | NA |
| Outcome data | 15* | *Cohort study*—Report numbers of outcome events or summary measures over time | NA | NA |
|  |  | *Case-control study—*Report numbers in each exposure category, or summary measures of exposure | NA | NA |
|  |  | *Cross-sectional study—*Report numbers of outcome events or summary measures | 17 | Done |
| Main results | 16 | *(*a) Give unadjusted estimates and, if applicable, confounder-adjusted estimates and their precision (eg, 95% confidence interval). Make clear which confounders were adjusted for and why they were included | 17-22 | This was a descriptive study with the goal of identifying associated with the outcome. No particular effect was being adjusted for. Therefore, only variables with important association with the outcome were retained. |
|  |  | (*b*) Report category boundaries when continuous variables were categorized | 17-22, Tables 2-4 | Done |
|  |  | (*c*) If relevant, consider translating estimates of relative risk into absolute risk for a meaningful time period | NA | NA |
| Other analyses | 17 | Report other analyses done—eg analyses of subgroups and interactions, and sensitivity analyses | 21-22 | Described in the results that three models were run. |
| Discussion | | |  |  |
| Key results | 18 | Summarise key results with reference to study objectives | 23-28 |  |
| Limitations | 19 | Discuss limitations of the study, taking into account sources of potential bias or imprecision. Discuss both direction and magnitude of any potential bias | 29 | Limitations were discusses. |
| Interpretation | 20 | Give a cautious overall interpretation of results considering objectives, limitations, multiplicity of analyses, results from similar studies, and other relevant evidence | 23-28 | Done |
| Generalisability | 21 | Discuss the generalisability (external validity) of the study results | 23 | The discussion states that the prevalence estimates obtained cannot be generalized to the three provinces nor to the country. However, the associations may be and are discussed in light of other studies. |
| Other information | | |  |  |
| Funding | 22 | Give the source of funding and the role of the funders for the present study and, if applicable, for the original study on which the present article is based | Financial disclosure section of the online submission system | See financial disclosure and competing interest statement. |

*Give information separately for cases and controls in case-control studies and, if applicable, for exposed and unexposed groups in cohort and cross-sectional studies.

**Note:** An Explanation and Elaboration article discusses each checklist item and gives methodological background and published examples of transparent reporting. The STROBE checklist is best used in conjunction with this article (freely available on the Web sites of PLoS Medicine at http://www.plosmedicine.org/, Annals of Internal Medicine at http://www.annals.org/, and Epidemiology at http://www.epidem.com/). Information on the STROBE Initiative is available at www.strobe-statement.org.
