# Supplementary figures and images for: Prevalence of and Factors Associated with Human Cysticercosis in 60 Villages in Three Provinces of Burkina Faso
Source: PLoS Negl Trop Dis. 2015 Nov 20;9(11):e0004248. doi: 10.1371/journal.pntd.0004248 (PMC4654529; doi:10.1371/journal.pntd.0004248)

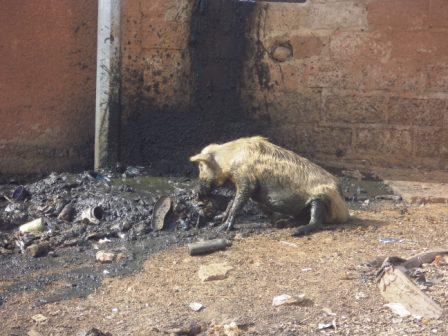

Supplement: S1 Fig — (JPG) [file pntd.0004248.s003.JPG]
